# Adhesive-Free Bonding of Monolithic Sapphire for Pressure Sensing in Extreme Environments

**DOI:** 10.3390/s18082712

**Published:** 2018-08-17

**Authors:** Jihaeng Yi

**Affiliations:** Department of Electronics and Electrical Engineering, Dankook University, Yongin-si 16890, Korea; jhyi@dankook.ac.kr; Tel.: +82-31-8005-3663

**Keywords:** adhesive-free sapphire bonding, pressure sensor, Fabry–Perot cavity, diaphragm deflection, white-light interferometry

## Abstract

This paper presents a monolithic sapphire pressure sensor that is constructed from two commercially available sapphire wafers through a combination of reactive-ion etching and wafer bonding. A Fabry–Perot (FP) cavity is sealed fully between the adhesive-free bonded sapphire wafers and thus acts as a pressure transducer. A combination of standard silica fiber, bonded sapphire wafers and free-space optics is proposed to couple the optical signal to the FP cavity of the sensor. The pressure in the FP cavity is measured by applying both white-light interferometry and diaphragm deflection theory over a range of 0.03 to 3.45 MPa at room temperature. With an all-sapphire configuration, the adhesive-free bonded sapphire sensor is expected to be suitable for in-situ pressure measurements in extreme harsh environments.

## 1. Introduction

The most widely used pressure sensors are based on semiconductor junctions, and their operation is restricted to environments under 150 °C. Sensors based on more advanced silicon-on-insulator (SOI) structures have extended the upper limit of their operating range up to 500 °C [1]. Recently, piezoresistive or capacitive pressure sensors have been constructed using silicon carbide (SiC); however, these are limited to a similar temperature range [2,3]. Optical interrogation techniques have developed the range of pressure sensor materials to include silica glass. Homogenous fused-silica sensor systems avoid any problems due to mismatched coefficients of thermal expansion (CTEs) and have the potential for miniaturization; such a sensor has been demonstrated and tested for operation up to 710 °C [4]. Silica glass sensors are ultimately restricted by dopant diffusion in the optical fiber, molecular creep of the amorphous glass, and softening of the glass membrane, all of which begin to occur below 1000 °C [5].

Advanced compound optical sensors have been attempted to push the thermal limit imposed by silica glass. Bonding of the high-temperature membrane is a major challenge to the development of this type of technology. One solution, which depends on anodic bonding between a SiC membrane and a silica glass body, has been suggested for achieving operation up to 1100 °C, and this sensor has been demonstrated to operate at 600 °C [6,7]. Anodic bonding is seen as a non-ideal solution because it requires that at least one material along the interface be silica glass, preventing the sensor from realizing the full high-temperature potential of its SiC diaphragm [8]. The operating temperature of any compound optical sensor is restricted by CTE mismatch, which can cause the structure to crack at high temperatures. 

To enable pressure sensing in harsh environmental conditions, homogeneous optical crystal-based structures that can operate in high-temperature regimes may provide the next step in the pressure sensor evolution. Sapphire (α-Al_2_O_3_) has a high melting point, excellent corrosion resistance, and low optical loss [9]. These properties make single-crystal sapphire an ideal candidate for optical sensors that could operate in harsh environments. Homogeneous sapphire temperature sensors have been shown to operate with good reliability [10,11,12]. Pure sapphire melts at over 2050 °C, and thin sapphire optical fibers have exhibited the ability to resist plastic deformation at temperatures over 1650 °C [13]. Unlike SiC and polycrystalline alumina, single-crystal sapphire displays negligible corrosion under high-temperature exposure to alkaline gases [14]. However, further sensor evolution is required to monitor pressures at temperatures up to 1600 °C in corrosive or oxidizing environments. 

In this paper, an adhesive-free bonded sapphire pressure sensor prototype is developed as a potential next-generation pressure sensor to provide reliable in-situ pressure measurements in harsh environments, allowing real-time monitoring close to the point where a reaction occurs. This direct pressure measurement capability will allow facility operators to monitor and optimize changing process conditions more precisely, resulting in more efficient energy production. Furthermore, direct pressure monitoring will eventually reduce the need for frequent system shutdowns by enabling more accurate failure prediction based on real operational data. 

## 2. Materials and Methods

### 2.1. Fabry–Perot (FP) Cavity Fabrication from Sapphire Wafer

Two 8 × 8 mm^2^ c-plane sapphire wafer pieces are the main constituents of the proposed pressure sensor. These elements are obtained by dicing a sapphire wafer (Meller Optics, Inc., Providence, RI, USA) that is epitaxially flat on one side (R_a_ ≤ 1 nm) and optically flat on the other side (R_a_ ≤ 5–10 µm). The diced sapphire wafers are permanently polished along the A-axis on the optically flat side. The permanent marking distinguishes the front side from the back side when the two wafer pieces are aligned before bonding. 

The fabrication procedure starts with inductively coupled plasma (ICP) etching, which creates a shallow cylindrical cavity on the sapphire wafer. The depth of the cavity is determined by the etching time and plasma composition. The cavity location and diameter are defined by a glass mask that exposes a 5 mm circular hole at the center of the cylindrical cavity. This mask is placed on the epitaxial side of the wafer, and the sample is etched using a Trion MiniLock reactive-ion etching (RIE) system with an ICP source. The etching agent is a mixture of 80% BCl_3_/20% Cl_3_ plasma formed under a total gas flow rate of 40 sccm, pressure of 10 mTorr, and ICP power of 300 W [15]. Under these conditions, the sapphire wafer is etched at a rate of 300 Å per minute. After 3 h of etching, a sapphire cavity is created with a depth of 4.8 µm at the deepest point.

The etched sapphire wafer is then bonded to a flat sapphire piece. The sealed hollow cavity thus formed can function as a pressure transducer, and the displacement of the diaphragm that encloses the cavity can be used to extract external pressure data [7,16]. Before bonding, the two wafer pieces, one of which includes the etched sensing cavity, are subjected to RCA cleaning and are then immersed in 85% H_3_PO_4_ at 150 °C for 45 min to remove any oxide remaining on their surfaces. Next, the wafers are immersed in diluted H_2_SO_4_ solution for 15 min to deposit a hydrophilic OH^−^ layer. The wafers are arranged such that the polished markings along their A-axes are aligned, and a weak pre-bond, based on hydrogen bonding, is formed by baking at 200 °C for 50 min. This pre-bonding step is performed using a clamping vise, which is intended to reduce any small gap at the wafer interface. A much stronger diffusion-based bond is formed by baking the wafer pieces at 1200 °C for over 50 h, during which time the wafers are compressed under a 700-g weight. Finally, the sample is annealed at 1200 °C, without any weight applied, to release any internal stresses trapped during bonding [16]. 

### 2.2. Pressure Sensing Test Experiment

**Figure 1:**
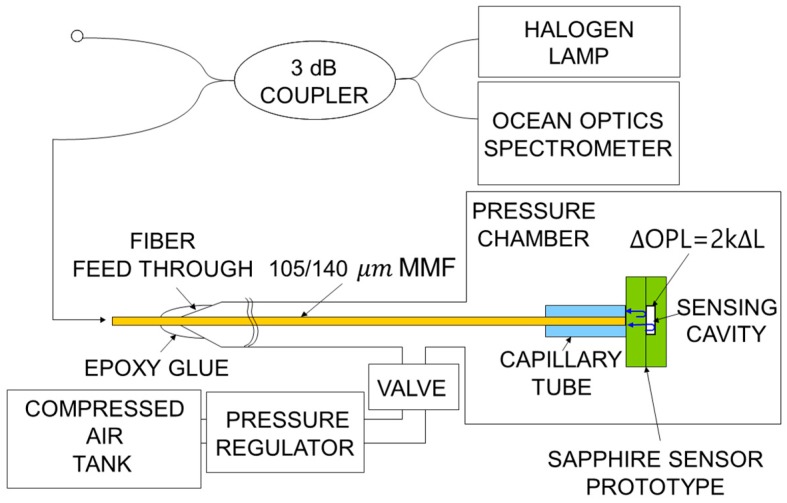
Schematic of pressure sensor test system. Broadband light from a halogen lamp is delivered through a Multi-Mode optical Fiber (MMF) through a 3-dB coupler and into the sapphire wafer.

To demonstrate the monolithic sapphire structure as a dynamic pressure sensor, a prototype optical interrogation system is constructed. For use in high-temperature applications, sapphire optical fiber provides an ideal mechanism to route the optical sensing signal to and from the wafer-based sensor. In a room-temperature evaluation of the sensor, a lead-in of 105/125-µm multimode silica optical fiber is chosen to approximate the highly multimode optical excitation passed through a sapphire wafer. The multimode lead-in fiber is cleaved and inserted into a silica ferrule and glued to the optically flat surface of the sapphire wafer pressure sensor, which is placed in a sealed chamber for testing, and pressure is controlled using a tank of compressed air and a pressure regulator, as shown in Figure 1. A needle valve is used to isolate the test chamber during collection of each data point to eliminate uncertainty due to drift of the pressure regulator. An Omegadyne analog pressure sensor, which has a +/−0.017 MPa accuracy over a range of 0 to 34.74 MPa, is monitored for sensor calibration and verification.

**Figure 2:**
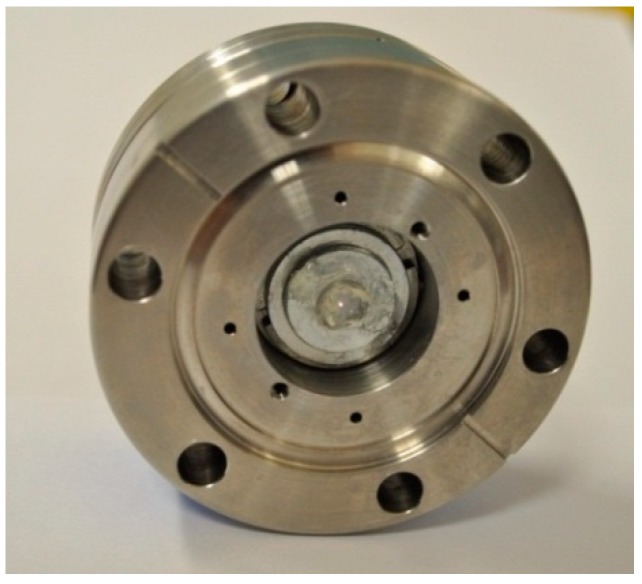
Bonded sapphire sample loaded in test chamber. Broadband light is focused on the center of the bonded sapphire wafer.

Sensor interrogation is performed using white-light interferometry, which has been proven to provide accurate measurements of physical distance [17]. The broadband light from a halogen lamp is delivered through the fiber and into the sapphire wafer through a 3-dB coupler, as illustrated in Figure 1. The lead-in fiber-optic beam focuses the broadband light down on the center of the direct-bonded sapphire wafer, as shown in Figure 2. The reflected spectrum, in which interference appears as a result of reflections from the two inner surfaces of the sensing cavity, is collected via an Ocean Optics USB spectrometer.

## 3. Results and Discussion

### 3.1. Sensor Fabrication and Diaphragm Deflection

**Figure 3:**
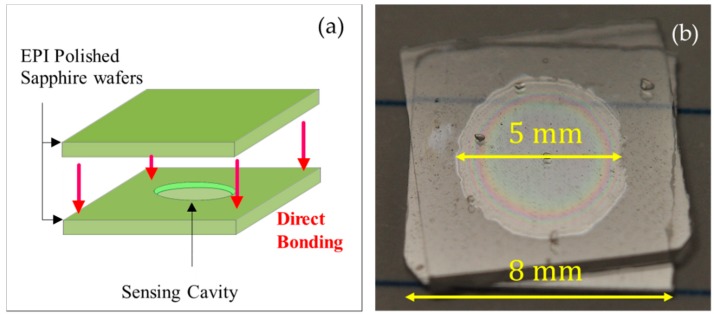
(**a**) Schematic of bonded sapphire wafer. (**b**) Photo of prototype sapphire sensor.

The adhesive-free bonding procedure applied to the two sapphire wafer pieces is illustrated in Figure 3a. A sapphire sensing cavity with a depth of 4.8 µm at the deepest point and a diameter of 5 mm is obtained as shown in Figure 3b. Because of the optical interference of the two narrow sapphire interfaces at the edge of the sensing cavity, we observe multiple colored Newton rings in these locations, which indicate a narrower spacing between two wafers [7]. In contrast, outside of the central cylindrical cavity, the sensor structure does not exhibit any interference rings, indicating that outside of the sensor cavity, the two sapphire pieces are in direct contact and are tightly bonded together. The observed Newton rings show that the bonding of two wafers is not tight at the edge of the cavity, compared with the bond outside of the sensor cavity. The tiny gap at the cavity edge is caused by the thermal expansion of the air inside the cavity during the pre-bonding and annealing processes. 

The misalignment, which is approximately 10° between the two sapphire wafers as shown in Figure 3b, is caused by the rotation of one of the sapphire wafers during the bonding process. However, this misalignment has no distinguishable influence on the direct bonding of the sapphire pieces and the operation of the pressure sensor, based on the results presented below. 

**Figure 4:**
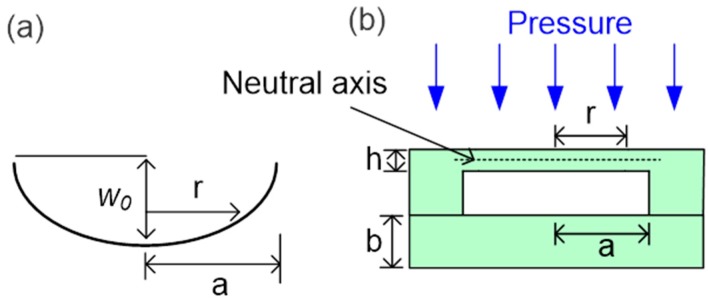
(**a**) Schematic of the small diaphragm deflection. (**b**) Schematic of the small deflection in the sensor cavity under uniform pressure: *b* is the thickness of the flat sapphire wafer, *a* is the maximum radius at which the deflection is measured, *h* is the thickness of the sapphire wafer containing the cavity, *r* is the radius from the center of the cavity at the measurement point, and *w*_0_ is the maximum deflection.

We consider the mechanical stress induced in the diaphragm that surrounds the sensing cavity under pressure, as illustrated in Figure 4. The dimensions of the sensing cavity significantly influence the pressure sensitivity and operating range of the sensor. At small deflections, there are no stresses on the neutral axis of the diaphragm; however, large deflections stress the outer surfaces of the diaphragm. One face experiences tensile stress, and the other experiences compressive stress at any given radius (*r*) from the center of the diaphragm. Therefore, radial and tangential stress components increase with increasing pressure and deflection radius and decrease with increasing diaphragm thickness, and a small diaphragm area experiences more stress than a larger area under the same pressure. The maximum values of the two stress components occur at the center of the diaphragm under large deflection [4]. By approximating the sensor diaphragm as a thin membrane, we can estimate its maximum deflection (*w*_0_) under external pressure with Equation (1):

(1) $$w_{0}=\frac{3\left(1-\nu^{2}\right)Pa^{4}}{8Eh^{3}}$$

where *P* is the external pressure, *a* is the maximum radius at which the deflection is measured, *E* is the Young’s modulus, *v* is the Poisson’s ratio, and *h* is the sapphire wafer thickness. The two sapphire wafers shown in Figure 3b are both 330 µm thick and are assumed to deflect identically; the diameter of the sensing cavity area is 5 mm. Therefore, the theoretical maximum deflection *w*_0_ is 3.90 µm at 3.45 MPa.

### 3.2. Fabry–Perot White-Light Interferometer and Sensor Interrogation

**Figure 5:**
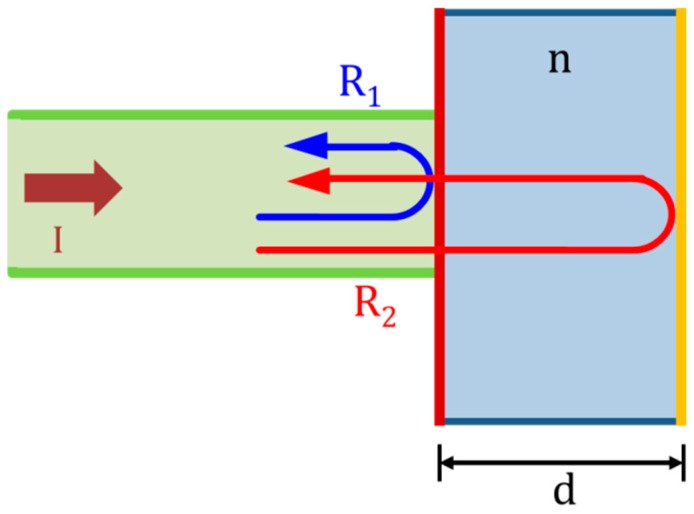
Schematic of the Fabry–Perot (FP) interferometric sensor: *I* is the normal incident light; the light reflects from two reflectors, *R*_1_ and *R*_2_; *n* is the refractive index of the cavity medium; and *d* is the depth of the air gap in the cavity.

Fabry–Perot white-light interferometry is a method used to interrogate the FP interferometric cavity at different wavelengths over a certain spectral range. The light from the source travels from the coupler to the sensor, which consists of two parallel reflectors as illustrated in Figure 5. A fraction of the normal incident light is reflected from approximately 7.6% of the sapphire-to-air interface, which is termed reflector-to-cavity reflection. A fraction of the light reflects at the first reflector and the remainder propagates to the FP cavity and reflects from the second reflector. Higher-order reflections from the cavity can be neglected because they have a low finesse, and the cavity can thus be approximated as a two-beam interferometer. The two reflections return to the detector through the same fiber and coupler. The sensor is thus designed so that an environmental variation can be effectively detected by monitoring the different optical path lengths of the two reflections.

The interference spectra can be normalized by removing the background spectrum of the light source $I_{0}\left(k_{n}\right)$ and can be expressed as:

(2) $$I_{N}\left(k_{n}\right)=\frac{I\left(k_{n}\right)}{I_{0}\left(k_{n}\right)}=A+Bcos\left(k_{n}\Delta L+\phi \right),$$

where $k_{n}$ = 2π/*λ* is the optical wavenumber, Δ*L* is the depth of the sensing cavity, $I_{0}\left(k_{n}\right)$ is the background spectrum of the broadband source, and ϕ is a constant phase term related to the multimode excitation of the FP cavity.

The spectra of the FP cavity are transformed by discrete Fourier transformation. The source spectrum $I_{0}\left(k\right)$ drops into the low-frequency region with spectral ranges (S) in the frequency domain. We can use a bandpass filter to separate the spectra to select the amplitude-modulated signal, $I_{0}\left(k\right)\cos\left(kL+\phi \right)$. The analytical model of the amplitude-modulated signal can be expressed as:

(3) $$I\left(k\right)=I_{0}\left(k\right)e^{\left(kL+\phi \right)},$$

where *k* = 2π/*λ* is the wave number, *L* is the optical path difference in the cavity, $I_{0}\left(k\right)$ is the spectrum of the incident light, and ϕ is the arbitrary initial phase difference between the two interfering optical waves. If two different spectral components of the source $\left(k_{1}\;\mathrm{and}\;k_{2}\right)$ are utilized, these two signal components then arrive at the spectrometer with different phases. The phase difference between these two spectral components is given by:

(4) $$L=2n\Delta L=\frac{\Delta \phi }{\left(k_{2}-k_{1}\right)},$$

where *k*_1,2_ = 2π/*λ*_1,2_, *n* is the refractive index of air (*n* = 1), and Δ*L* is the depth of the sensing cavity. We use a few special points with fixed phase relations by monitoring the spectral locations of the peaks or valleys in the interference spectrum. When the phase difference, ∆*φ*, between two adjacent peaks (or valleys) becomes 2π, the absolute value of the cavity air gap length, Δ*L*, can be obtained by applying Equation (4).

We select and normalize the single band of the amplitude-modulated signal from Equation (2) by using a single-band filter, which can be implemented by a double-band filter followed by a Hilbert transform. The filtering and Hilbert transformation can be calculated efficiently by fast Fourier transformation. We then fit the sinusoidal curve of the initial (reference) spectrum with the reflected spectrum, whereby we can obtain the optical path difference, *L*, using Equation (4) when the phase difference, ∆*φ*, becomes 2π. From the value of *L*, we can then uniquely determine the applied external pressure that matches the data based on Equation (2). Sensor interrogation is conducted by white-light interferometry. The spectrum of light reflected by the sensor is measured using an Ocean Optics spectrometer. 

**Figure 6:**
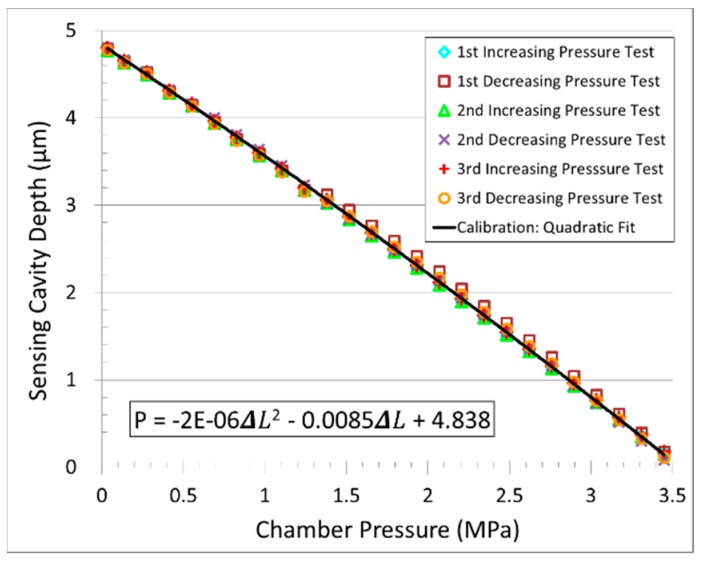
Three cycles of dynamic pressure testing and calibration of sensor prototype.

The dynamic sensor response is investigated by increasing and decreasing the pressure over a range of 0.03 to 3.45 MPa for three cycles, as shown in Figure 6. The sensor responds almost linearly over this pressure range, and hysteresis is below the level of measurement resolution. After each cycle, the sensor reverts to the same initial cavity length of 4.8 µm +/−0.008 µm at atmospheric pressure. However, there is no physical model, only a second-order polynomial. Thus, the good agreement between the experimental results and the regression is trivial. A calibration curve is fitted to the sensor data via a quadratic regression (R^2^ = 0.998) and is compared with data from an electronic pressure gauge. The theoretical model fits well with the experimental results. The pressure can be determined from the calibration curve, which is linear. Thus, the applied external pressure can be determined from the value of the sensing cavity depth, Δ*L*.

**Figure 7:**
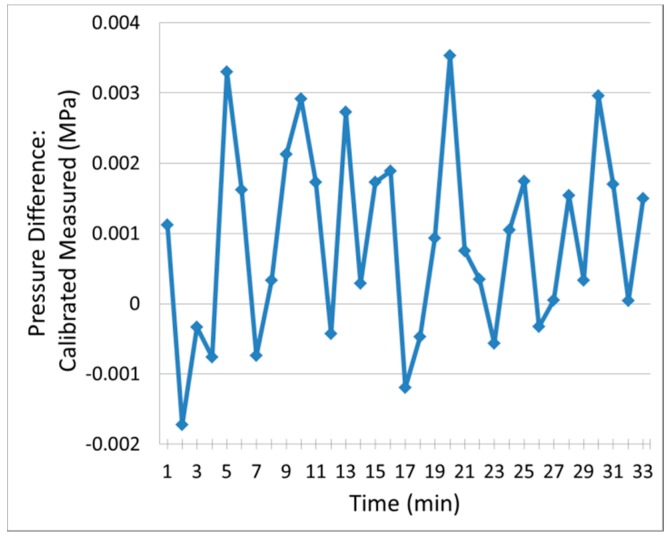
Sensor resolution measurement: data taken in 1-min intervals at constant pressure with the chamber pressure maintained at 1.39 MPa.

Sensor resolution is measured by checking the standard deviation of the calibrated sapphire sensor output at constant pressure, as shown in Figure 7. A small leak in the test chamber is compensated using pressure data measured by the electronic gauge. A total of 33 data points are obtained at 1-min intervals, with the chamber pressure maintained at 1.39 MPa. A sensor resolution of +/−0.003 MPa (2σ) is measured, which is equivalent to 0.19% of the tested range. Given that the accuracy of the electronic pressure gauge is only 0.017 MPa, it is possible that the sensor has an even higher resolution.

**Figure 8:**
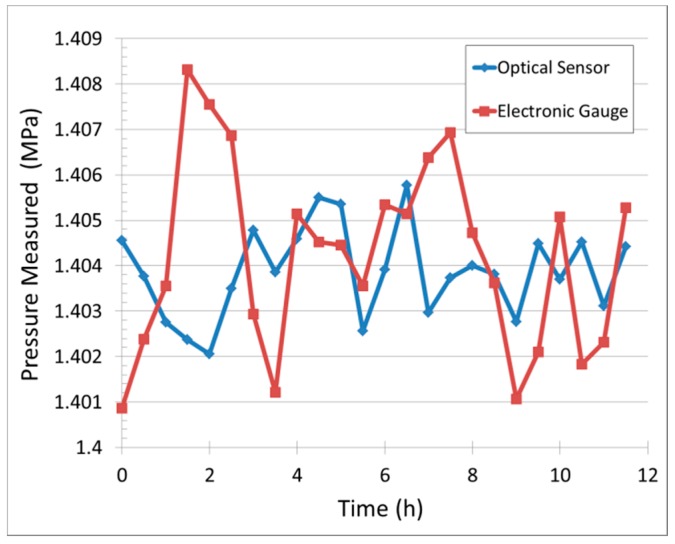
Sensing cavity leakage test: data taken at constant pressure over 12 h.

The chamber is maintained at 1.404 MPa for 12 h, and additional data points are taken every 30 min, as shown in Figure 8. Throughout all measurement cycles, the results indicate no observable deviation beyond the level of measurement resolution. Cumulatively, these results show that the sensor cavity is tightly sealed without any air leakage, and the proposed sensor can be used as a dynamic pressure sensor.

## 4. Conclusions

This paper presented the fabrication of a monolithic sapphire pressure sensor through cavity etching and sealed diaphragm bonding. A preliminary optical test was performed to measure the resolution of the structure and verify its potential for use as a dynamic pressure sensor. The operation of the sensor produced a linear response, with a resolution of at least +/−0.003 MPa (0.19%) and negligible hysteresis over a 0.03 to 3.45 MPa range. The results of long-term testing at constant pressure clearly indicate that the sensing cavity was entirely sealed for the full duration of the test, confirming the high-quality bond between the two wafer surfaces. This monolithic pressure sensor, made entirely of sapphire, is thus free from CTE mismatch and the associated adhesive breakdown problems that plague other sensors. Through appropriate selection of the etched sensing cavity diameter, the dynamic range of the sensor can be tuned to operate over almost any range of pressures. If fully developed with sapphire lead-in fiber and high-temperature packaging, the proposed sapphire sensor could be used in a variety of applications in the energy and transportation industries, enabling dynamic measurement of pressure in harsh environments, where such measurements have never been possible before.

## References

[1] Kurtz A.D., Ned A.A., Goodman S., Epstein A.H. Latest ruggedized high temperature piezoresistive transducers. Proceedings of the NASA 2003 Propulsion Measurement Sensor Development Workshop.

[2] Ziermann R., Von Berg J., Obermeier E., Wischmeyer F., Niemann E., Möller H., Eickhoff M., Krötz G. (1999). High temperature piezoresistive β-sic-on-soi pressure sensor with on chip sic thermistor. Mat. Sci. Eng. B.

[3] Eickhoff M., Möller H., Kroetz G., Berg J.v., Ziermann R. (1999). A high temperature pressure sensor prepared by selective deposition of cubic silicon carbide on soi substrates. Sens. Actuators A Phys..

[4] Zhu Y., Cooper K.L., Pickrell G.R., Wang A. (2006). High-temperature fiber-tip pressure sensor. J. Lightw. Technol..

[5] Wang A., Gollapudi S., Murphy K.A., May R.G., Claus R.O. (1992). Sapphire-fiber-based intrinsic fabry–perot interferometer. Opt. Lett..

[6] Pulliam W.J., Russler P.M., Fielder R.S. High-temperature high-bandwidth fiber optic mems pressure-sensor technology for turbine engine component testing. Proceedings of the Environmental and Industrial Sensing.

[7] Pulliam W.J., Russler P.M., Mlcak R., Murphy K.A., Kozikowski C.L. Micromachined sic fiber optic pressure sensors for high-temperature aerospace applications. Proceedings of the Environmental and Industrial Sensing.

[8] Iyer S.S. Silicon Wafer Bonding Technology for VLSI and Mems Applications (Emis Processing Series, 1). https://skl35cffj07.storage.googleapis.com/MDg1Mjk2MDM5NQ==07.pdf.

[9] Merberg G.N., Harrington J.A. (1993). Optical and mechanical properties of single-crystal sapphire optical fibers. Appl. Opt..

[10] Huang Z., Pickrell G., Xu J., Wang Y., Zhang Y., Wang A. Sapphire temperature sensor coal gasifier field test. Proceedings of the Sensors for Harsh Environments.

[11] Cooper K.L., Wang A., Pickrell G.R. (2006). Optical Fiber High Temperature Sensor Instrumentation for Energy Intensive Industries.

[12] Wang A., Wang G.Z., Gollapudi S., May R.G., Murphy K.A., Claus R.O. Advances in sapphire optical fiber sensors. Proceedings of the Fiber Optic Smart Structures and Skins V.

[13] Tong L., Shen Y., Chen F., Ye L. (2000). Plastic bending of sapphire fibers for infrared sensing and power-delivery applications. Appl. Opt..

[14] Pickrell G.R. High-Temperature Alkali Corrosion Kinetics of Low-Expansion Ceramics. https://elibrary.ru/item.asp?id=5634942.

[15] Peng W., Qi B., Pickrell G., Wang A. Investigation on cubic zirconia-based pressure sensor for high temperature application. Proceedings of the Sensors 2003.

[16] Peng W., Pickrell G.R., Wang A. (2005). High-temperature fiber optic cubic-zirconia pressure sensor. Opt. Eng..

[17] Ferber M., Ogle J., Tennery V., Henson T. (1985). Characterization of corrosion mechanisms occurring in a sintered sic exposed to basic coal slags. J. Am. Ceram. Soc..

